# Immobilisation and Epidural Anaesthesia in a Eurasian Lynx (*Lynx lynx*) Undergoing Pelvic Limb Orthopaedic Surgery

**DOI:** 10.1155/2024/6373424

**Published:** 2024-08-09

**Authors:** Sonia Campos, Pierre Picavet, Olivier Bertrand, Charlotte Sandersen, Alexandru Tutunaru

**Affiliations:** ^1^ CECAV-Veterinary and Animal Research Centre Faculty of Veterinary Medicine Lusófona University, Lisbon, Portugal; ^2^ University Veterinary Clinic Faculty of Veterinary Medicine Liége University, Liége, Belgium; ^3^ Wildlife Park of La Roche-en-Ardenne, Namur, Belgium

**Keywords:** anaesthesia, epidural, lidocaine, lynx, morphine

## Abstract

Immobilisation and anaesthesia of wild felids may be complex and potentially dangerous events, making it difficult to implement more advanced anaesthetic techniques such as neuraxial anaesthesia. A Eurasian lynx was referred for femur fracture repair after it was seen with lameness of the left pelvic limb sustained in its natural environment. The animal was remotely darted using a combination of ketamine (5 mg/kg) and xylazine (5 mg/kg) intramuscularly. Once immobilised, the lynx was transported to the veterinary hospital in a restraining cage. After induction and endotracheal intubation, pelvic limb radiographs confirmed a closed, comminuted fracture of the left femur that required open reduction and internal stabilisation. A sacrococcygeal epidural was performed before surgery using lidocaine (2 mg/kg) and morphine (0.1 mg/kg) to complement the ketamine–xylazine–isoflurane anaesthesia, which allows a low-end-tidal isoflurane concentration. Clinical signs were continuously monitored and remained stable during the entire procedure, with the exception of a temperature that decreased to 35.8°C. No intraoperative analgesic rescues were necessary. Recovery was smooth and uneventful. The lynx showed no signs of motor weakness after surgery or other side effects related to the anaesthetic procedure. The successful management of this surgical case suggests that the described anaesthetic protocol could be recommended in orthopaedic procedures of the pelvic limbs in wild Felidae.

## 1. Introduction

The nature of wild animals dictates that any restraint and handling demand chemical immobilisation, which can be challenging for both the animal and personnel [1]. In most cases, anaesthetic combinations have been extrapolated from protocols used in domestic species and scarce information is known, especially with regard to drug pharmacokinetics and pharmacodynamics [2]. Many anaesthetic combinations for wild felid restraint can be found in the literature for medical and research purposes [3, 4]. Most anaesthetic protocols include a cyclohexamine anaesthetic agent and an *α*_2_-adrenoreceptor agonist, though benzodiazepines, opioids, or a combination of these are also described [5–7]. Although not an endangered species, the preservation of the *Lynx lynx* is of growing interest; consequently, improvements in anaesthesia and surgical techniques are essential [5]. Epidural anaesthesia is one of the local anaesthesia techniques most studied and performed in both human and small animal practice but is often underused in zoo and exotic settings [3, 8]. The performance of regional anaesthesia, such as neuraxial blocks, can be difficult to accomplish as capture and restraint of the animal are required, as well as transportation of the anaesthetised patient to a hospital setting. The use of epidural techniques has been described in tigers [9], cheetahs [10], and leopards [11] for pelvic limb surgery and in lions for abdominal surgery [12]. Currently, there are no reports about its application to the lynx species. The injection of a local anaesthetic in the epidural space will block the spinal nerve roots in a volume- and concentration-dependent way [13]. There is a great variety of drugs and combinations that may be administered in the epidural space [14]. The most commonly used are local anaesthetic agents such as lidocaine, bupivacaine, and ropivacaine, which differ in their onset, duration of action, and systemic side effects [8, 15]. Epidural administration of opioids, *α*_2_-adrenoreceptor agonists, and dissociative anaesthetics may also be used in combination with local anaesthetics to provide intra- and postoperative analgesia [16]. Opioid drugs administered epidurally provide effective pain relief, long duration of action, minimal motor blockade, and limited adverse effects. They are suitable for epidural administration because they cross the dura mater and bind to the opioid receptors in the dorsal horn of the spinal cord [14, 16, 17]. As mammals, wildlife felids may also benefit from epidural anaesthesia for surgery on fractured pelvic limbs. This study describes chemical immobilisation, epidural anaesthesia technique, and anaesthetic management in a Eurasian lynx with a femur fracture.

## 2. Case Description

An adult female Eurasian lynx (*Lynx lynx*) weighing 16.6 kg and approximately 2 years old was referred to the small animal hospital at the Faculty of Veterinary Medicine of Liège University. The animal had a 4-day history of lameness and suspicion of a limb fracture. The lynx presented lameness on a 5 over 5 (based on an ordinal visual analogue scale defined from 0 to 5; 0—*no visual lameness*; 5—*nonweight-bearing lameness*) of the left pelvic limb. Before transportation, the animal was immobilised using a combination of xylazine (5 mg/kg) and ketamine (5 mg/kg) darted intramuscularly by means of a remote delivery drug system (Dan-Inject blowgun projector [Dan-Inject Model IM, CO_2_ injection pistol, Denmark]). This combination was prepared using 500 mg of lyophilized xylazine (Rompum Droge Stof, Bayer, Belgium) that was dissolved in 5 mL of ketamine hydrochloride (Nimatek Vet. 100 mg/mL, Dechra, Netherlands), obtaining a final concentration of 100 mg/mL of each substance. From this main solution, 0.8 mL was withdrawn and diluted in 2 mL of water for injections, making a final volume of 2.8 mL for injection. The projector contained a 3.5-mL dart syringe (S300, Dan-Inject compressed air, Denmark) with a 1.5 × 25 mm plain needle with a red stabiliser. Once the animal had stopped in a stable position, the lightweight dart was shot from a 13-m distance estimated by a laser range finder, aiming for the large muscle masses of the right thoracic limb. Immobilisation was satisfactory with only one shot, and the animal was transported in a cage to the teaching hospital under continuous monitoring of breathing, ocular reflexes, and peripheral haemoglobin oxygen saturation (SpO_2_) (Pulse Oximeter Edan H100B, Edan Instruments, France). Transport lasted 50 min, clinical signs remained stable, and there was no need for resedation.

**Figure 1:**
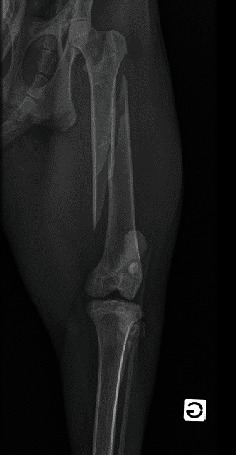
Ventrodorsal radiography of the left femur showing a closed comminuted mid-diaphyseal spiral fracture and soft tissue swelling of the surrounding muscles.

Upon arrival at the hospital, the lynx remained nonresponsive to external stimuli and cardiopulmonary stable. An intravenous cannula 20G (Vasofix Safety, BBraun, Germany) was placed in the right cephalic vein, and blood was collected for haematology (Table 1), biochemistry, and ion analysis (Table 2). Presurgical bloodwork revealed a slight increased total protein concentration (TP = 7.8 g/dL), possibly due to dehydration [18]. A physical examination under sedation (1 h and 35 min after chemical restraint) showed a heart rate (HR) of 101 beats/min, a respiratory rate (RR) of 10 breaths/min with no abnormal sounds on cardiopulmonary auscultation, an arterial blood pressure of 98 mmHg as measured by Doppler (Doppler Vet BP, Mano Médical, France), and a temperature (T) of 36.6°C (slight hypothermia). Face mask preoxygenation before induction was performed with 100% oxygen (flow = 4 L/min) for 5 min. Anaesthesia was induced using 0.2 mg/kg of midazolam hydrochloride (Dormazolam, Dechra, Belgium) and 3.5 mg/kg of propofol to effect (Propovet, Zoetis, Belgium), both IV. The trachea was intubated with the lynx in the sternal position using a cuffed endotracheal tube (7.5 mm ID endotracheal PVC tube, Kruuse, Belgium) and a Miller-Size 4 laryngoscope. Two puffs of lidocaine 2% spray (Intubeaze, Dechra, Netherlands) were previously applied to the arytenoids to minimise laryngospasm. Anaesthesia was maintained with isoflurane (IsoFlo 100%, Zoetis, Belgium) vaporised in 100% oxygen (flow = 1.2 L/min) using a coaxial rebreathing system (end-tidal isoflurane between 1.0% and 1.2%). At this point, the animal was taken to imaging for radiographs of the pelvic limbs. Radiographs confirmed a comminuted fracture with a spiral fracture line on the left femoral shaft. Lateral–proximal and cranial displacement of the distal fracture end was also noted, along with soft tissue swelling of the surrounding muscles (Figure 1). During this period, the animal had end-tidal carbon dioxide partial pressure (EtCO_2_), SpO_2_, HR, and RR monitored using a portable multiparametric monitor (Prizm 3TM Charmcare, Seoul, Korea), and arterial blood pressure using an ultrasonic Doppler device applied to the palmar artery and a sphygmomanometer with a proper-sized cuff placed in the correspondent mid-antebrachium. Vital signs remained within the normal range during image acquisition. Preoperative preparation of the patient continued in the preparation room with clipping for the epidural anaesthesia procedure and placement of a 22G (Vasofix Safety, BBraun, Germany) cannula at the right dorsal metatarsal artery for invasive blood pressure (IBP) monitoring. The epidural technique was performed with the lynx in lateral recumbency with the surgical site as the dependent site, avoiding further trauma to the fractured bone. After a strict aseptic technique, an epidural needle with Tuohy bevel 18G (Perican®, Bbraun, Germany) was used to approach the sacrococcygeal space (sacrum and first coccygeal vertebrae). The anatomic references were the ones normally used for small cats' epidurals, with the needle positioned between the caudal edge of the sacrum and the first coccygeal vertebrae after identifying the injection site by moving the tail up and down while palpating the sacrococcygeal region [8, 15]. The confirmation of the correct positioning of the needle was based on the “popping sensation,” absence of blood or cerebrospinal fluid aspiration, and lack of resistance to the injection of a test dose because of the subatmospheric pressure of the epidural space. A combination of preservative-free morphine hydrochloride 0.1 mg/kg (Morphine Sterop 10 mg/mL, Sterop, Bruxelles) and lidocaine hydrochloride 2 mg/kg (Linisol 2%, BBraun, Germany), diluted in sterile NaCl 0.9% at a final volume of 0.2 mL/kg, was injected at a velocity of 2 mL/min [15]. These doses were based on data collected from wild felids [10, 12]. The animal remained in lateral recumbency with the surgical site on the dependent site for 30 min, allowing migration/action of epidurally administered drugs (gravity effect) while surgical preparations proceeded. Electrocardiography (ECG), IBP, and RR were continuously monitored during the epidural anaesthesia procedure and remained within the normal range. Cefazolin 20 mg/kg (Cefazolin Sandoz 1 g, Novartis, Belgium) was slowly administered IV, 30 min before surgery, and repeated every 90 min intraoperatively.

**Figure 2:**
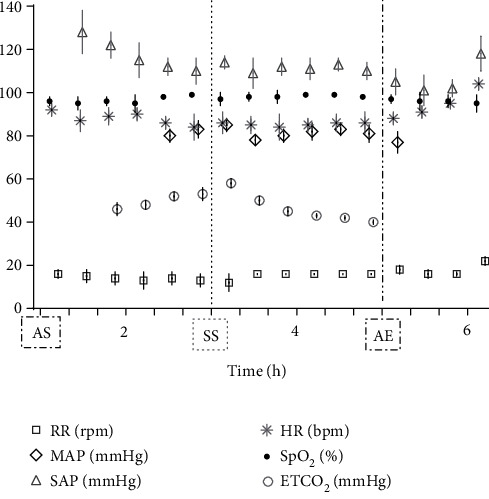
Cardiopulmonary values (mean ± SD) of the anaesthetized lynx (*Lynx lynx*) during transportation, diagnostics, surgery, and recovery periods. For each variable (RR, MAP, SAP, HR, SPO_2_, and ETCO_2_), the standard deviation is represented by the vertical line crossing the symbol. Specific time points are identified: AS, anaesthesia start; SS, surgery start; AE, end of anaesthesia.

**Figure 3:**
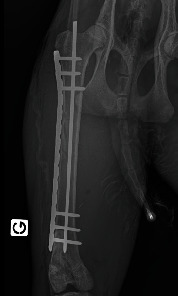
Postsurgery ventrodorsal view of the left femur fracture reduction showing placement of a 3 mm intramedullary pin and one locking plate (14 holes) with six locked screws (three proximal and three distal to the fracture line).

During instrumentation, the animal was allowed to breathe spontaneously and clinical signs were closely monitored. In the surgery room, the animal was positioned in lateral right recumbency and was connected to the mechanical ventilator (Penlon Prima 320 Anaesthesia Station, InterMed, United Kingdom) for intermittent positive pressure mechanical ventilation, as hypercapnia (ETCO_2_ = 62 mmHg) was observed. The initial settings were tidal volume of 10 mL/kg, RR of 18 breaths/min, and peak inspiratory pressure of 12 cmH_2_O in order to maintain normocapnia (ETCO_2_ 35–45 mmHg). No changes in these settings were made during the surgical time. The arterial cannula was connected to a pressure transducer, which had been zeroed to atmospheric pressure. The transducer was positioned at the level of the right atrium. Lactated Ringer's solution (Vetivex, Dechra, Netherlands) was infused intravenously throughout the entire anaesthesia at a rate of 5 mL/kg/h. Monitoring during surgery included ECG, IBP, SpO_2_, oesophageal T, side stream capnography, and measurement of inspired and expired concentrations of oxygen and isoflurane (VT-9000, Veterinary Techniques Int., Netherlands). All data were collected every 5 min. Cardiopulmonary values during the anaesthetic period are represented in Figure 2. The lynx showed great anaesthetic stability during surgery, on 0.7%–1.0% end-tidal isoflurane concentration, with no palpebral (but corneal reflex present) and ventromedial positioned ocular globe. HR varied between 85 and 107 beats/min, SAP between 98 and 126 mmHg, and MAP around 69–80 mmHg. SpO_2_ fluctuated between 94% and 99%. The inspired fraction of oxygen was 90%–70%. Capillary refill time (CRT) was inferior to 2 s, and mucous membrane colour was pink during the entire period. The lowest T observed was 35.8°C immediately before the beginning of surgery. The animal was rewarmed using a heating pad and a forced-air warming device (Bair Hugger, 3 M, United Kingdom) that covered most of its body. The T increased gradually to 37.5°C (0.5° per 40 min). There were no intraoperative surgical or anaesthetic complications. Rescue analgesia was not required, as the patient showed no signs of nociception. A lateral approach to the left femur was performed. Osteosynthesis was made using a 3 mm centromedullary pin and one locking compression plate (LCP) with six locked screws, the two proximal ones being monocortical. Suture closing was done by the conventional plane-by-plane approach. At the end of the surgical procedure, the animal returned to imaging, which confirmed the correct realignment of the operated femur (Figure 3). The total time of anaesthesia was 6 h and 20 min, and the surgery time was 1 h and 40 min. The patient was then moved to the transport cage, all cannulas were removed, and meloxicam 0.1 mg/kg (Metacam 5 mg/mL, Boehringer Ingelheim, Belgium) was given subcutaneously. Extubation was performed when the lynx started to have mandibular tonus, approximately 1 h and 10 min after the end of anaesthesia. Recovery from anaesthesia was gentle and uneventful. The lynx returned shortly thereafter to the wildlife park where it was allowed to recover in a flat area to ensure strict rest. It became ambulatory the same day. Meloxicam was continued orally for 3 days at a dose of 0.05 mg/kg hidden in food. The lynx was allowed to recover in a smaller area of the wild park to reduce physical effort. Six weeks later, the animal was no longer lame and was released into the wild.

## 3. Discussion

This report is aimed at describing the chemical restrain, epidural anaesthesia technique, and anaesthetic management of a femur fracture repair of a lynx.

Wildlife anaesthesia can be a challenge due to the defensive behaviour of animals and the stress associated with manipulation. Proper planning prior to capture and anaesthesia is essential for both the animal and the personnel involved [3]. There are few data on the use of drug combinations to immobilise *Lynx lynx* individuals in the wild, as reports on anaesthetic dosage, response, and effectiveness are scarce [19]. Allometric extrapolation of drug doses from domestic cats may be ineffective and unpredictable due to their small body size [2]. In addition, free-ranging felids often require higher drug doses to achieve immobilisation than captive animals [20]. Therefore, reporting different anaesthetic protocols provides an opportunity to manipulate animals more safely and document data that can support species conservation, such as in the *Lynx lynx* [5].

The use of sedatives, anaesthetic drugs, and immobilisation techniques has been described for restraint and anaesthesia in wildlife felids [3]. Immobilisation and physical restraint techniques include remote darting, traps, squeeze or baited cages, and manual restraint of smaller and docile animals [21]. Remote darting drug devices have become an essential tool for low-stress capture and safe drug delivery of medication in nondomesticated and uncooperative species, with animals over 15 kg of particular interest [4]. Rifles, dart guns, or blowpipes are the most commonly used devices and can be selected based on the target's distance, aiming at the large muscle area of the thoracic and pelvic limbs [21]. In this case report, immobilisation using a CO_2_-powered dart gun containing an anaesthetic combination of drugs was chosen for animal and human safety concerns, and it also showed to be the least time-consuming method given the urgency of the medical issue. There is a vast literature description about the use of anaesthetics in wild felids [3, 4, 20]. The choice of drugs is often dictated by the availability and cost, drug combination effects and high therapeutic index, length and purpose of the procedure, as well as the health status of the animal. Most documented protocols include combinations of dissociative agents with *α*_2_-adrenergic receptor agonists, benzodiazepines, and/or opioids for synergistic effects and smoother recoveries [3]. Protocols with a sole agent are described but no longer recommended due to the very high dose requirement which may also show more serious side effects [22]. When determining a drug dosage, compared to domestic cats, the wild felid may require a higher dose [2]. Reported doses of ketamine can range from 11 to 44 mg/kg when administered alone, or 4–26 mg/kg combined with 1–4 mg/kg xylazine IM in small felids [1–3]. Rockhill et al. evaluated the effectiveness of medetomidine and butorphanol as substitutes for xylazine in ketamine-based field immobilisation protocols for *Felis rufus* [19]. They concluded that both protocols provided safe and reliable sedation despite the benefits of using a multimodal protocol that allowed the decrease of ketamine doses and, as a consequence, faster recoveries. In a case report describing surgical correction of traumatic patellar luxation in a Eurasian lynx, the authors used dexmedetomidine, methadone, and midazolam intramuscularly with good anaesthetic stability and outcome [6]. The same was reported in another case for diaphragmatic peritoneal pericardial hernia repair in the Eurasian lynx with medetomidine, ketamine, and butorphanol [23]. The intranasal administration of the zolazepam–tiletamine combination was also described with success in a Eurasian lynx presented with a neck wound [7]. In none of these reports, locoregional anaesthesia was performed and the vaporiser setting of isoflurane was 3% in the first case [6] and up to 2.4% of sevoflurane in the second [23] for anaesthetic maintenance. The xylazine–ketamine immobilisation protocol used in our case was adapted by the professionals of the wildlife park who were familiar with both drugs' effects. However, the dose of xylazine may have been slightly overestimated when compared to the described data [2], most likely due to weight miscalculation at the darting. The induction protocol comprised the administration of the short-acting hypnotic propofol in conjunction with the benzodiazepine midazolam given to effect, which is in alignment with previous data [6], and provided gentle hypnosis and relaxation that allowed endotracheal intubation. Other induction protocols in the lynx are described, such as the use of propofol alone [23] and a face mask with sevoflurane in oxygen [5]. Up to date, there are no data available on the use of alfaxalone alone or in combination in lynx. During maintenance of anaesthesia, the lynx showed great clinical stability and no signs of nociception, as clinical parameters (HR, SAP, MAP, SpO_2_, and EtCO_2_) remained within the normal range during the entire procedure. This is of particular interest as the amount of expired isoflurane was ≤ 1% and there was no need for analgesic rescues, highlighting the MAC-sparing effect of the premedication and local anaesthesia applied. The main disadvantage of the ketamine–xylazine implemented protocol, which was also documented by others, was a slightly prolonged recovery (70 min) [19]. The decision to not antagonise xylazine with an *α*_2_-adrenergic antagonists was based on the cardiovascular stability of the animal during the recovery period and to minimise the stress related to transport. Moreover, yohimbine, a selective *α*_2_-adrenergic antagonist used to reverse xylazine sedative effects, was not a commercially available option (unlicensed product) on the day of surgery.

This case report also describes the successful performance and outcome of a sacrococcygeal epidural for a femur fracture repair in a lynx. The provision of epidural anaesthesia in small animals for orthopaedic surgery of the pelvic limb or caesarean section has been documented for over 70 years [8]. The epidural administration of analgesics and local anaesthetics reduces the need for other anaesthetics and analgesic rescues, promotes muscle relaxation, decreases the likelihood of adverse effects associated with the systemic administration of opioids, and improves recovery [14]. All these benefits were observed in the lynx of this study. First, the amount of isoflurane needed for the maintenance of anaesthesia was reduced after the epidural injection which is in agreement with previous data [9, 10]. Secondly, it allowed great intraoperative anaesthetic stability, muscle relaxation, and fast fracture reduction without the requirement of analgesic rescues. Finally, the lynx recovered shortly and well after anaesthesia and locomotion was normal 6 weeks postoperatively, as expected [24]. The cephalad spread and effectiveness of the block are affected by the dose, which is related to the concentration and volume of the injectate [13]. Lidocaine, bupivacaine, and ropivacaine are the most common local anaesthetics used by neuraxial routes [14]. The different agents, their properties, and their potential effects when given extradurally are reviewed elsewhere [15]. In our case, the drugs chosen for epidural anaesthesia were lidocaine and morphine. On the one hand, lidocaine promotes analgesic and physiochemical properties (such as high lipid solubility and low protein binding capacity compared to other drugs), a short onset of action, and a short duration of motor blockade. On the other hand, morphine is the least lipophilic of the opioids and, therefore, has the slowest onset of action (up to 60 min) but is capable of obtunding nociception up to 24 h without significant effect on motor function and minimal systemic uptake [14, 16]. The affinity of opioid drugs for their receptors in the spinal cord is increased by local anaesthetics, so the combination of opioids with local anaesthetics has been proven beneficial [15, 25]. Although synergism and enhancement of analgesia were aimed at the conjunction of the abovementioned agents, the nonimpairment of the motor function was of great concern in this particular case, as the animal would have to become ambulatory once it arrived in its natural environment. This was the main reason why the sacrococcygeal epidural technique was chosen, as was the volume of injectate administered (0.2 mL/kg), and a local anaesthetic with a longer duration of action, such as bupivacaine, was avoided. Additionally, as in felids, the dural sac may extend caudally into the body of the S1 vertebra, and performing a lumbosacral epidural would increase the chances of doing a spinal blockade or damage [13].

Finally, no iatrogenic side effects or complications were observed either by the immobilisation protocol or the epidural anaesthesia, though many are described in the literature, such as vomiting, bradycardia, arrhythmias, hypotension, ataxia, seizures, or even cardiac arrest [3, 14]. Another important limitation of this case study is related to the unavailability of the ultrasound machine/electrical nerve stimulator device to perform sciatic and femoral nerve blocks, as it would be an excellent alternative to local pain relief without any motor impairment of the other limb.

In conclusion, the capture and immobilisation of a lynx by intramuscular darting with a ketamine–xylazine combination were safely accomplished. The sacrococcygeal epidural injection of 2 mg/kg of lidocaine combined with 0.1 mg/kg of morphine can be considered easy to perform, providing effective intraoperative analgesia, reducing the isoflurane concentration requirements to maintain anaesthesia, and preventing postoperative motor blockade. Therefore, neuraxial anaesthesia can be suggested as part of a balanced anaesthesia protocol for surgeries of the pelvic limb in the lynx.

## Figures and Tables

**Table 1:** Haematological parameters of the lynx before the surgery.

**Parameter**	**Value**	**Normal range** ^a^
Erythrocytes (×10^12^/L)	8.43	4.3–7.8
Haemoglobin (g/L)	129	84–140
Haematocrit (%)	42	26–46
Mean corpuscular volume (fL)	50	56–62
Mean corpuscular haemoglobin concentration (g/L)	306	290–310
Leucocytes (×10^9^/L)	9.17	5–12.2
Neutrophils (×10^9^/L)	7.99	7–18
Lymphocytes (×10^9^/L)	0.69	10–30
Monocytes (×10^9^/L)	0.48	0–5
Eosinophils (×10^9^/L)	0	0–2
Platelets (×10^9^/L)	437	336–502

^a^Lynx data obtained from Weaver and Johnson [18].

**Table 2:** Biochemistry and ion values of the lynx before the surgery.

**Parameter**	**Value**	**Normal range** ^a^
BUN (mg/dL)	31	28–46
Creatinine (mg/dL)	2.3	1.5–3.3
Total proteins (mg/dL)	7.8	6.4–7.6
Albumin (mg/dL)	3.8	3.3–3.9
AST (IU)	24	20–32
ALT (IU)	35	23–46
Glucose (mg/dL)	154	79–136
K^+^ (mEq/L)	4.7	3.4–5.6^b^
Na^+^ (mEq/L)	151	147–156^b^
Cl^−^ (mEq/L)	109	99–110^b^

^a^Lynx data obtained from Weaver and Johnson [18].

^b^Reference range from domestic cats.

## Data Availability

All data is present within the manuscript.
